# Preparation of Low-Defect Manganese-Based Prussian Blue Cathode Materials with Cubic Structure for Sodium-Ion Batteries via Coprecipitation Method

**DOI:** 10.3390/molecules28217267

**Published:** 2023-10-25

**Authors:** Xinyu Dong, Haifeng Wang, Jiawei Wang, Qian Wang, Hao Wang, Wenhao Hao, Fanghai Lu

**Affiliations:** 1 School of Materials and Metallurgy, Guizhou University, Guiyang 550025, China; dxy799376151@126.com (X.D.); mm.hfwang@163.com (H.W.); wang_qian0509@163.com (Q.W.);; 2 Guizhou Provincial Engineering Technology Research Center of Manganese Materials for Batteries, Tongren 554300, China; 3 Guizhou Provincial Key Laboratory of Metallurgical Engineering and Energy Saving, Guiyang 550025, China; 4 School of Materials and Energy Engineering, Guizhou Institute of Technology, Guiyang 550002, China

**Keywords:** Prussian blue analogs, sodium-ion battery, cathode materials, electrochemical performances

## Abstract

Sodium-ion batteries have important application prospects in large-scale energy storage due to their advantages, such as safety, affordability, and abundant resources. Prussian blue analogs (PBAs) have a stable and open framework structure, making them a very promising cathode material. However, high-performance manganese-based Prussian blue cathode materials for sodium-ion batteries still suffer from significant challenges due to several key issues, such as a high number of vacancy defects and a high crystal water content. This article investigates the effects of the Fe-Mn molar ratio, Mn ion concentration, and reaction time on the electrochemical performance of MnHCF during the coprecipitation process. When Fe:Mn = 1:2, c(Mn^2+^) = 0.02 mol/L, and the reaction time is 12 h, the content of interstitial water molecules in the sample is low, and the Fe(CN)_6_ defects are few. At 0.1 C, the prepared electrode has a high initial discharge specific capacity (121.9 mAh g^−1^), and after 100 cycles at 0.2 C, the capacity retention rate is 65% (~76.2 mAh g^−1^). Meanwhile, the sample electrode exhibits excellent reversibility. The discharge capacity can still be maintained at around 75% when the magnification is restored from 5 C to 0.1 C. The improvement in performance is mainly attributed to two aspects: On the one hand, reducing the Fe(CN)_6_ defects and crystal water content is conducive to the diffusion and stable structure of N. On the other hand, reducing the reaction rate can significantly delay the crystallization of materials and optimize the nucleation process.

## 1. Introduction

With the rapid development of the new energy industry and the urgent need for low-carbon living, the efficient utilization of renewable new energy technologies requires low-cost and long-lived energy storage systems [1,2]. Sodium-ion batteries (SIBs) not only have the characteristics of abundant raw material reserves and low prices but also have electrochemical behaviors similar to lithium-ion batteries. They can be used as a supplement to lithium-ion batteries (LIBs) for large-scale energy storage and are considered the most promising electrochemical energy storage technology [3,4,5,6]. However, the main challenges faced by SIBs are the need to develop low-cost, high-specific-capacity, and long-cycle-life cathode materials. Prussian blue analogs (PBAs), compared with other SIB cathode materials, such as layered oxides and polyanionic compounds, have an open three-dimensional channel structure and a face-centered cubic structure. PABs are very promising cathode materials for energy storage, due to their advantages of a high specific capacity, stable electrochemical performance, environmental friendliness, and low cost [7,8,9,10,11]. The general formula of PBAs can be described as Na_x_M[Fe(CN)_6_]_1-y_□y nH_2_O (0 < x < 2, 0 < y < 1), where M is a transition metal element, such as Fe, Mn, Co, Ni, and Cu, and □ represents [Fe(CN)_6_] vacancies [12,13,14,15,16]. When M is Mn, it is generally referred to as manganese-based ferrocyanide (MnHCF). However, this material generally suffers from a poor rate performance and cycle life due to problems such as crystal water, low electronic conductivity, and the effect of Jahn Teller with Mn^3+^ [17,18,19,20,21]. For example, Huang et al. coated stable nickel-based Prussian blue (NiHCF) on the surface of MnHCF, forming a MnHCF/NiHCF core–shell structure, extending the cycle life. However, the inertness of Ni has a certain impact on the specific capacity of the material [22]. Peng et al. introduced strong chelating agents in the coprecipitation reaction to improve the particle size, lattice integrity, and cyclic stability of the product. However, strong chelating agents have the challenges of high costs and difficulty in mass production [23]. Peng et al. designed a nanoreactor using the ball milling solid-phase method to successfully prepare high-specific-capacity MnHCF products without the need for additives. However, the ball milling method still has serious problems with agglomeration [24]. In summary, changes in various conditions during the preparation process may result in more vacancies and a higher coordination water content in Fe(CN)_6_. The prepared materials are prone to irreversible structural transformation during the charging and discharging process, resulting in a low capacity utilization and poor cycling stability.

Herein, this research systematically investigated the effects of different Fe:Mn and Mn ion concentrations and reaction times on the final product composition, morphology, and electrochemical performance. Based on traditional preparation methods, the inorganic chelating agent thiourea was added to delay crystallization and suppress defect formation, thoroughly optimizing the preparation process of this material.

## 2. Results and Discussions

### 2.1. Effect of Fe-Mn Ratio on Properties of MnHCF

**Figure 1:**
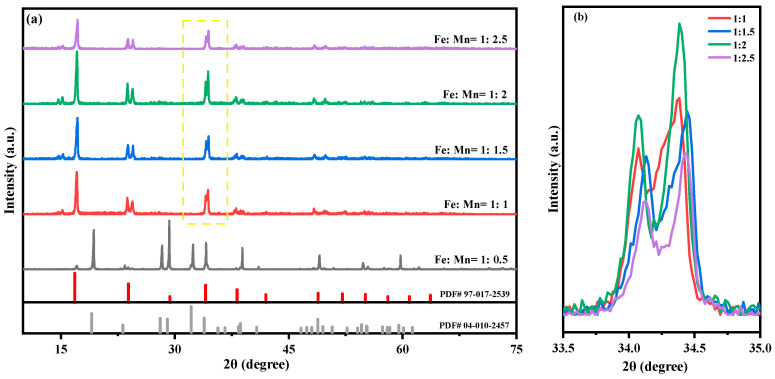
XRD patterns of MnHCF prepared with different Fe-Mn molar ratios: (**a**) XRD spectra of MnHCF; (**b**) partial enlarged view of XRD in the yellow box (2θ = 33.5°–35°).

According to the chemical formula of Na_2_MnFe(CN)_6_, the Fe-Mn molar ratios are set to 1:0.5, 1:1, 1:1.5, 1:2, and 1:2.5, respectively, in order to study the effect of raw materials on the performance of the sample at ratios less than, equal to, and greater than the theoretical ratio. The structure of the prepared MnHCF material is tested using XRD, and the results are shown in Figure 1. The results show that the sample can only be successfully prepared when Fe:Mn > 1:0.5; otherwise, the product is Na_2_SO_4_ precipitated from the solution (PDF # 04-010-2457). The prepared sample corresponds well to the characteristic diffraction peaks on the PDF standard card, with a high diffraction peak intensity, clear peak positions, and no other impurity peaks, indicating that the synthesized series of materials have very pure phases and high crystallinity. The splitting peak near 24.3° and 34.3° of the material indicates that the prepared MnHCF material has a monoclinic phase structure (spatial group P21/n). Compared with the relevant literature, it can be seen that materials with a monoclinic structure have a higher sodium content, which is more conducive to improving the specific capacity of the material [25,26]. Through the partial enlarged view of Figure 1, it can be seen that the successfully prepared samples exhibit different peaks near 34.3°. The sample with Fe:Mn = 1:2 has a higher peak intensity and more pronounced peak separation compared to other samples, indicating a higher degree of crystallinity and better structural integrity of the material. When continuing to increase the molar ratio content of Mn, excessive manganese can lead to a decrease in the crystallinity of the sample, but the effect is not good.

**Figure 2:**
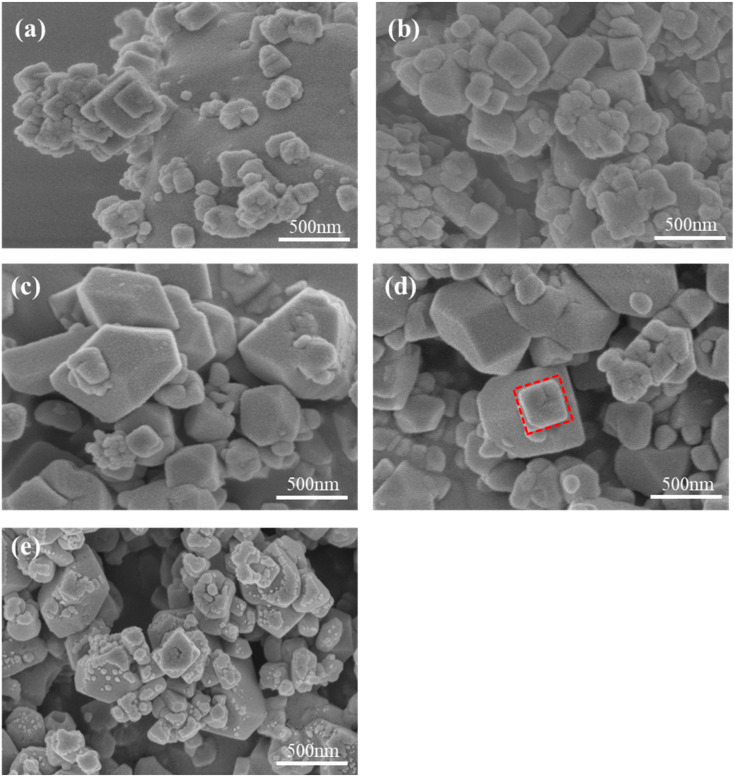
SEM images of MnHCF prepared with different Fe-Mn molar ratios: (**a**) Fe:Mn = 1:0.5, (**b**) Fe:Mn = 1:1, (**c**) Fe:Mn = 1:1.5, (**d**) Fe:Mn = 1:2, and (**e**) Fe:Mn = 1:2.5.

The morphology characteristics of MnHCF materials prepared with different Fe-Mn molar ratios are observed using SEM. As shown in Figure 2, all samples exhibit irregular shapes, which is related to the high asymmetry of the monoclinic phase structure shown by XRD results. The particle size of the sample ranges from tens of nanometers to hundreds of nanometers. This result indicates that a large number of lattice vacancies and coordinated water not only lead to structural defects but also to morphological defects and irregularities. When Fe:Mn reaches 1:2, the overall shape of the sample particles changes from irregular to regular cubic, with some particles showing clear cubic shapes. This indirectly reflects that increasing the Mn content can promote the improvement of the material’s structural integrity. However, if the Fe-Mn ratio continues to increase, excessive manganese will lead to rapid nucleation and insufficient growth of the sample crystal, resulting in the formation of small particles attached to the surface of large particles, which is not conducive to the electrochemical performance of the material.

**Figure 3:**
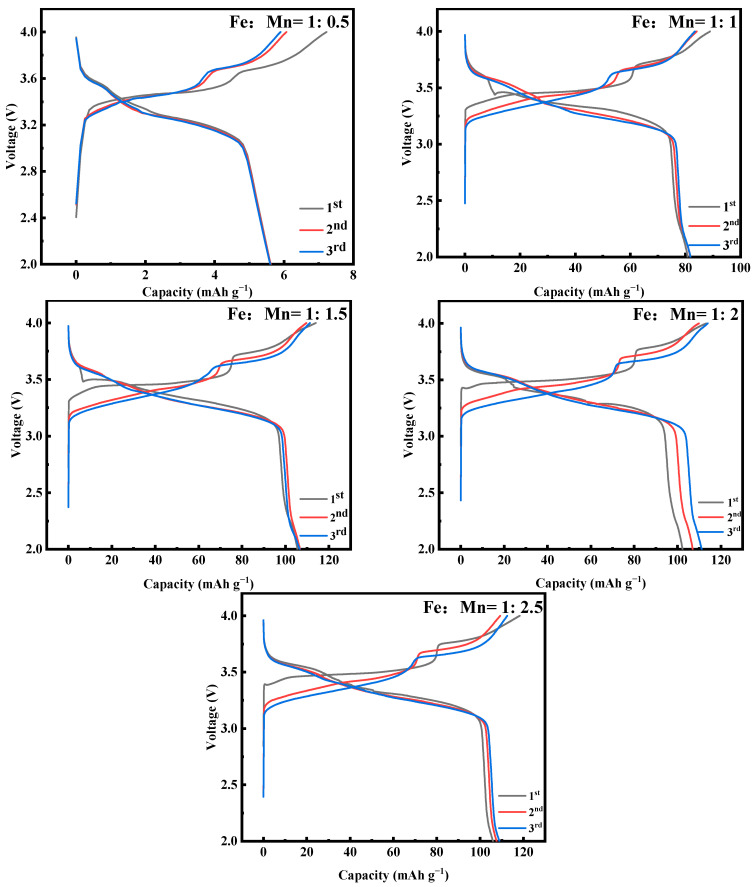
Charge–discharge curves of MnHCF prepared with different Fe-Mn molar ratios at 0.1 C for the first three cycles.

**Figure 4:**
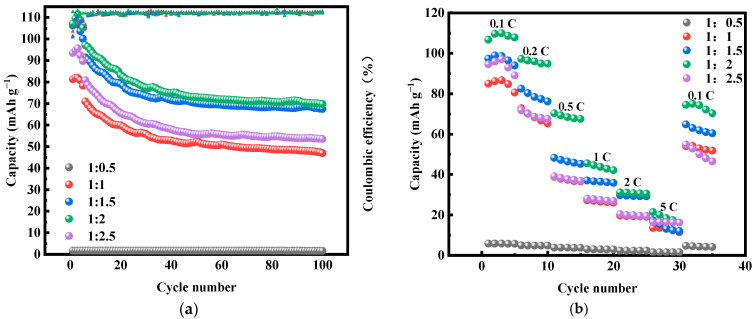
Cyclic and rate performance of MnHCF prepared with different Fe-Mn molar ratios: (**a**) cyclic performance and coulombic efficiency of MnHCF prepared with different Fe-Mn molar ratios at 0.2 C; (**b**) rate performance of MnHCF prepared with different Fe-Mn molar ratios.

Figure 3 shows the first three constant-current charging and discharging experiments of MnHCF electrode materials prepared with different Fe-Mn molar ratios under test conditions of 0.1 C and a voltage range of 2.0–4.0 V. As shown in the figure, when Fe:Mn = 1:0.5, a small amount of Mn addition cannot prepare MnHCF materials, which corresponds to the XRD results. The materials successfully prepared have two distinct platforms at 3.65/3.42 V and 3.9/3.77 V, which correspond to two redox reactions of Mn^2+^/Mn^3+^ and Fe^2+^/Fe^3+^, respectively. When Fe:Mn = 1:2, the initial discharge capacity of MnHCF is 111.1 mAhg^−1^, which is equivalent to 75% of the theoretical capacity and corresponds to a high initial Coulombic efficiency of 97.5%. The loss of initial Coulombic efficiency can be attributed to the side reaction of crystal water decomposition during the charging process. However, the cyclic performance of MnHCF with a monoclinic structure is not excellent, with a capacity retention rate of about 50% after 100 cycles at 0.2 C. Among the five samples, the MnHCF electrode prepared with Fe:Mn = 1:2 exhibits a relatively excellent cycling performance, with a retention rate of 61% after 100 cycles at 0.2 C (Figure 4a) and good stability. At a high current density of 1 C, the MnHCF electrode prepared with Fe:Mn = 1:2 exhibits a high capacity retention rate of 55% (Figure 4b), which is much higher than other samples. Meanwhile, when the sample recovers from 5 C to 0.1 C, the discharge capacity of the MnHCF electrode prepared with Fe:Mn = 1:2 can still be maintained at around 70% (Figure 4b), indicating good stability and reversibility at different rate performances. According to the relevant literature, the differences in the electrochemical performances of these materials are mainly caused by two aspects: (1) A large number of Fe(CN)_6_ vacancy defects. These defects will be occupied by some coordination water, leading to an increase in the lattice water content and blockage of Na^+^ transport channels [21,27,28,29,30]. At the same time, the presence of excessive defect sites and coordination water leads to the collapse of the cyanide connection framework during the cycling process, further affecting the storage performance of sodium ions. (2) The reaction rate is too fast. An excessively fast reaction rate will accelerate the precipitation generation rate, leading to the formation of particles of an uneven size and irregular shape in the sample. It is closely related to the sodium content and lattice defects of the product.

**Figure 5:**
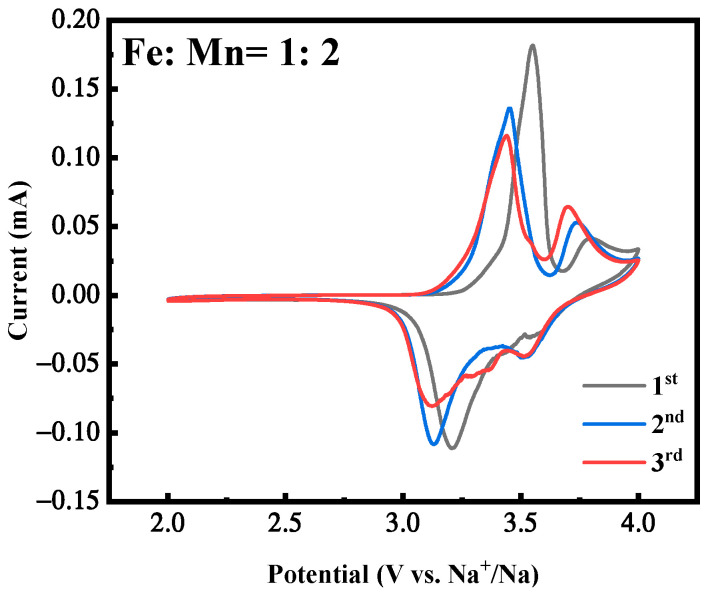
CV curves of MnHCF prepared with Fe:Mn = 1:2.

Figure 5 is the cyclic voltammetry curves of the first three cycles of the MnHCF electrode with the best electrochemical performance under Fe:Mn = 1:2 preparation conditions, with a voltage range of 2.0~4.0 V and a scanning rate of 0.1 mV/s. It can be seen that the first circle of the CV curve exhibits two obvious oxidation peaks, corresponding to voltages of approximately 3.5 V and 3.7 V, respectively, corresponding to the oxidation process of Fe^2+^ → Fe^3+^ and Mn^2+^ → Mn^3+^ [31,32,33,34]. There are two reduction peaks between 3.3 V and 3.8 V. One is a strong reduction peak, corresponding to the change in Fe ions from +3 to +2. In addition, there is a weak reduction peak, which corresponds to the reduction in Mn ions from +3 to +2. In addition, the CV curves of the first three cycles indicate that the material exhibits a certain degree of reversibility during cycling, corresponding to and consistent with the plateau of its constant-current charge–discharge curve.

**Figure 6:**
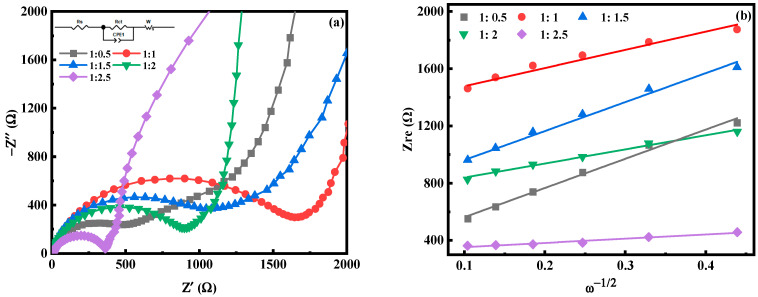
EIS spectra of MnHCF prepared with different Fe-Mn ratios: (**a**) EIS spectra; (**b**) linear relationship of Z_re_ and ω^−1/2^ of MnHCF electrodes prepared with different Fe-Mn ratios.

Figure 6 is the electrochemical impedance spectroscopy (EIS) test diagram of MnHCF electrodes prepared with different Fe-Mn molar ratios. The illustration in Figure 6a is the fitted equivalent circuit diagram. Each electrode appears as a semicircle in the high-frequency region, which is caused by the charge transfer process; in the low-frequency region, it appears as an inclined straight line, which is attributed to the Warburg diffusion process [35,36,37]. In the equivalent circuit, R_s_ and R_ct_ represent the electrolyte resistance and charge transfer resistance of the electrochemical system, respectively, corresponding to the semicircle in the high-frequency region. The CPE constant phase component represents a double-layer capacitor, where Z_w_ typically represents the diffusion resistance of the sodium ions within the electrode, corresponding to the diagonal line in the low-frequency region. It can be seen that the Rct of the electrode is relatively large, which is consistent with the low conductivity of the MnHCF material. Obviously, the R_ct_ value of the electrode prepared at Fe:Mn = 1:2 is relatively small among the five samples (Table 1), indicating the rapid reaction kinetics of Na^+^ diffusion between the electrode and electrolyte interface. In addition, the diffusion coefficient (D_Na_) of sodium ions can be calculated from the slope line (Figure 6) in the low-frequency region, according to the following formula [38]:

D_Na_ = R^2^T^2^/2A^2^n^4^F^4^C_Na_^2^σ^2^

where R is the gas constant, T is the absolute temperature, A is the surface product of the positive electrode, n is the number of electrons transferred during the redox process per molecule, F is the Faraday constant, C is the concentration of Na^+^ in the electrolyte, and σ is the Warburg factor. For MnHCF materials prepared at Fe:Mn = 1:2 and 1:2.5, the D_Na_ value is significantly higher than other samples. This indicates that these two materials can better promote charge transfers and sodium ion diffusion at the electrode–electrolyte interface. However, due to the high Mn ion content and fast reaction rate of the material prepared at Fe:Mn = 1:2.5, the large number of small particles generated can shorten the transport path of Na ions and improve the diffusion rate, but they are more likely to cause the dissolution and collapse of Mn ions, leading to a decrease in the electrochemical performance. In summary, when Fe:Mn = 1:2, it can effectively adjust the morphology and structure of the material, enabling it to exhibit a better electrochemical performance.

### 2.2. The Effect of Mn Ion Concentration on the Performance of MnHCF

**Figure 7:**
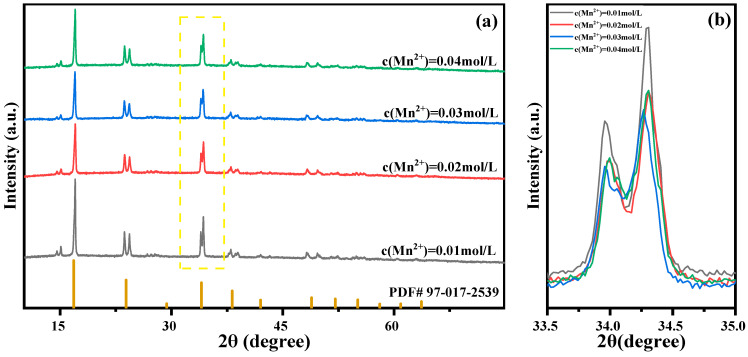
XRD patterns of MnHCF prepared with different Mn ion concentrations: (**a**) XRD spectra of MnHCF; (**b**) partial enlarged view of XRD in the yellow box. (2θ = 33.5°–35°).

Solutions were configured with Mn ion concentrations of 0.01 mol/L, 0.02 mol/L, 0.03 mol/L, and 0.04 mol/L for reactions to study the effect of Mn ion concentration changes on the sample performance. The structure of the prepared MnHCF material was tested using XRD, and the results are shown in Figure 7. The results show that MnHCF prepared under different Mn ion concentrations has the same monoclinic crystal structure (spatial group P21/n). Narrow and sharp diffraction peaks appear near 2θ of 14.3°, 16.6°, 23.8°, and 33.8°, indicating that the prepared MnHCF has good crystallinity. Meanwhile, as the concentration of Mn ions decreases, the intensity of the diffraction peak gradually increases, indicating that the crystallinity of the sample is better when the concentration of Mn ions is lower.

**Figure 8:**
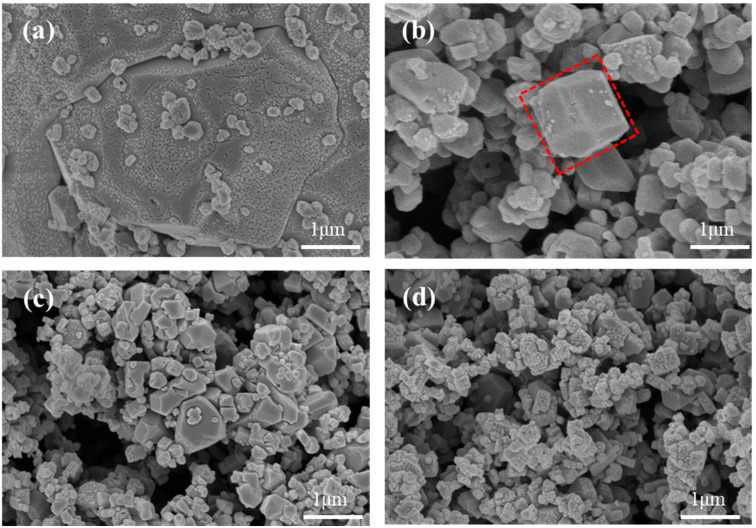
SEM images of MnHCF prepared with different Mn ion concentrations: (**a**) c(Mn^2+^) = 0.01 mol/L, (**b**) c(Mn^2+^) = 0.02 mol/L, (**c**) c(Mn^2+^) = 0.03 mol/L, and (**d**) c(Mn^2+^) = 0.04 mol/L.

Figure 8 shows the SEM images of MnHCF samples prepared with different Mn ion concentrations. Obviously, a higher concentration of Mn ions will result in a faster precipitation rate, smaller average particle size, and significant agglomeration of particles. Therefore, reducing the concentration of Mn ions can effectively reduce the nucleation rate of nanoparticles and promote the formation of a regular cubic structure. It is worth noting that when the concentration of Mn ions decreases to 0.01 mol/L, the primary particles begin to aggregate into larger secondary particles, with a particle size of 5 μ. An excessive particle radius is not conducive to the transfer of Na^+^ within the material, thereby affecting the electrochemical performance of the material. From the figure, it can be seen that when the concentration of Mn ions is 0.02 mol/L, the morphology of the sample begins to show relatively complete cubic particles, indicating a significant improvement in the regularity of the particle structure of the material.

**Figure 9:**
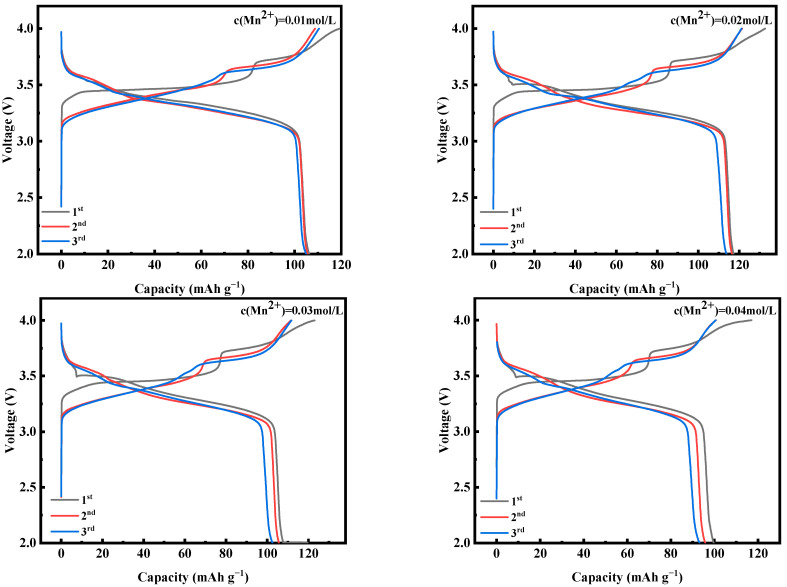
Charge–discharge curves of MnHCF prepared with different Mn ion concentrations at 0.1 C for the first three cycles.

**Figure 10:**
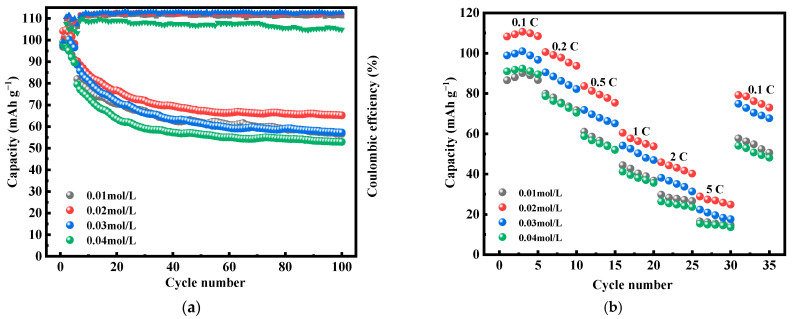
Cyclic and rate performance of MnHCF prepared with different Mn ion concentrations: (**a**) cyclic performance and coulombic efficiency of MnHCF prepared with different Mn ion concentrations at 0.2 C; (**b**) rate performance of MnHCF prepared with different Mn ion concentrations.

Figure 9 shows the first three constant-current charging and discharging experiments of MnHCF electrode materials prepared with different Mn ion concentrations under test conditions of 0.1 C and a voltage range of 2.0–4.0 V. From the figure, it can be seen that the electrode material with the Mn ion concentration of 0.02 mol/L has a larger discharge specific capacity, which can reach 117.3 mAh g^−1^. Figure 10a shows a comparison of the cyclic performance of MnHCF electrode materials prepared with different Mn ion concentrations at 0.2 C. It can be seen that the sample exhibits a higher initial discharge specific capacity when the Mn ion concentration is 0.02 mol/L, and the subsequent capacity decrease is relatively slow. The discharge capacity of the battery decreased to 66 mAh g^−1^ after 100 cycles, with a capacity retention rate of 63%. Figure 10b is a comparison chart of the rate performances of samples prepared with different Mn ion concentrations at different currents. It can be seen that the discharge specific capacity of the electrodes prepared with Mn ion concentrations of 0.02 mol/L at different rates is higher than that of other samples, indicating a better rate performance. When the discharge capacity of the material is restored from 5 C to 0.1 C, it can recover to 75%, indicating good reversibility.

**Figure 11:**
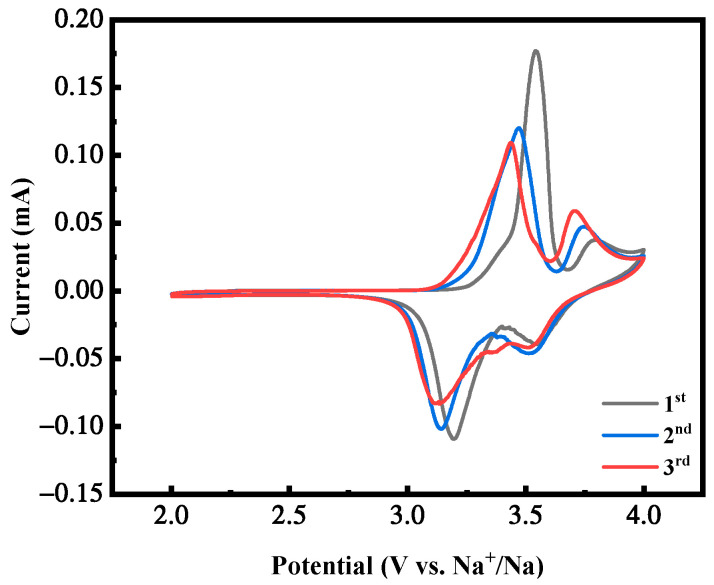
CV curves of MnHCF prepared with Mn ion concentration of 0.02 mol/L.

Figure 11 shows the cyclic voltammetry curves of the first three cycles of the MnHCF electrode with the best electrochemical performance under the condition of a Mn ion concentration of 0.02 mol/L, with a voltage range of 2.0–4.0 V and a scanning rate of 0.1 mV/s. As the cycle progresses, the shape of the CV peak of the sample remains almost unchanged, with only a slight decrease in intensity, indicating that the two redox centers, Fe and Mn, remain structurally intact and maintain stable electrochemical activity. This curve trend indicates that the reaction of the battery during the cycling process is reversible, corresponding to and consistent with the plateau of its constant-current charging and discharging curve.

**Figure 12:**
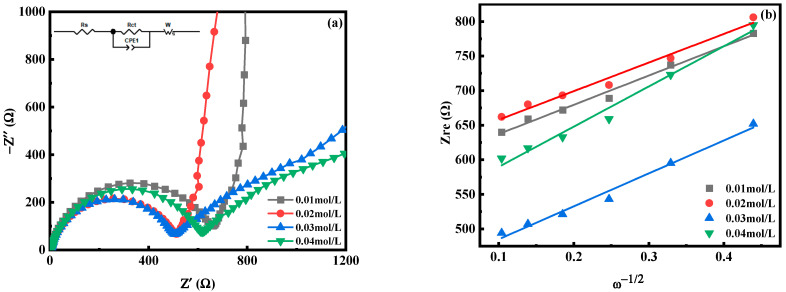
EIS spectra of MnHCF prepared with different Mn ion concentrations: (**a**) EIS spectra; (**b**) linear relationship of Z_re_ and ω^−1/2^ of MnHCF electrodes prepared with different Mn ion concentrations.

Figure 12 is the electrochemical impedance spectroscopy (EIS) test diagram of MnHCF electrodes prepared with different Mn ion concentrations, and the illustration in Figure 12a is the fitted equivalent circuit diagram. The electrochemical impedance spectra of electrodes prepared from different samples are composed of oblique lines in the low-frequency region and semicircles in the high-frequency region, belonging to typical Nyquist and Warburg impedances. The semicircle region corresponds to the electron transfer impedance R_ct_ at the interface between the electrode material and the electrolyte. The R_ct_(480.1 Ω) of the electrode prepared at c(Mn^2+^) = 0.02 mol/L is smaller than that of other materials, indicating that the material has a faster diffusion rate of Na^+^ from the electrolyte to the electrode interface and has a fast charge transfer ability. As shown in Table 2, the diffusion coefficient of Na^+^ in electrodes prepared with different materials can also be calculated. The D_Na_ of the electrode material prepared at c(Mn^2+^) = 0.02 mol/L is significantly higher than that of other materials, indicating an improvement in the kinetics of Na^+^ desorption and a faster diffusion rate of Na^+^ under this condition. In summary, appropriately reducing the concentration of Mn ions can improve the electrochemical performance of the material, which is consistent with the previous analysis results.

### 2.3. Effect of Reaction Time on the Performance of MnHCF

**Figure 13:**
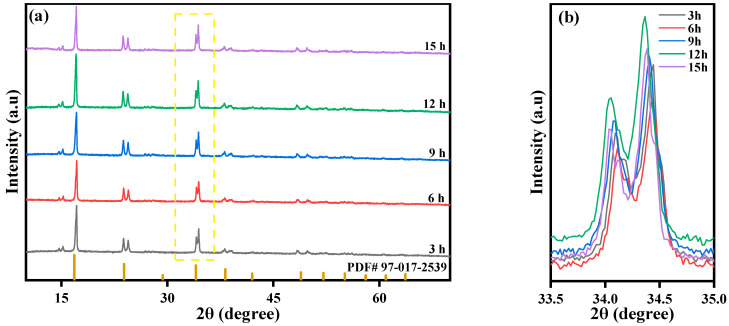
XRD patterns of MnHCF prepared at different reaction times: (**a**) XRD spectra of MnHCF; (**b**) partial enlarged view of XRD in the yellow box. (2θ = 33.5°–35°).

Under fixed reaction conditions, the Fe-Mn molar ratio is 1:2 and the Mn ion concentration is 0.02 mol/L. The reaction time is controlled to be 3 h, 6 h, 9 h, 12 h, and 15 h by controlling the droplet acceleration of solution B. The XRD peaks of these five materials prepared at different reaction times are shown in Figure 13. The overall similarity of each material indicates that the five materials have the same crystal structure (spatial group P21/n) and all possess the crystal characteristics of Prussian blue materials. The XRD diffraction peaks of the five samples appeared at 2θ of 14°, 16°, 24°, 34°, and 37°, with each diffraction peak being sharp, indicating good crystallinity. At the same time, the prepared samples had no obvious impurity peaks, indicating high purity and few impurities. The sample prepared with a reaction time of 12 h has the highest peak strength and the best crystallinity.

**Figure 14:**
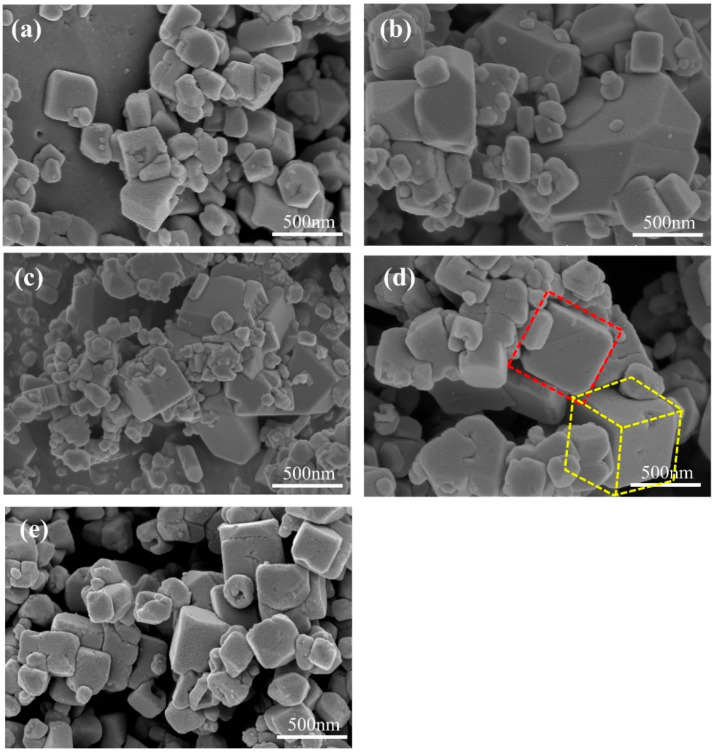
SEM images of MnHCF prepared at different reaction times: (**a**) 3 h, (**b**) 6 h, (**c**) 9 h, (**d**) 12 h, (**e**) 15 h.

The SEM images of samples prepared at different reaction times are shown in Figure 14. When the reaction time is shorter than 3 h, the generated particles have two distinct sizes. The smaller particles are significantly agglomerated and attached to the larger particles. This indicates that the reaction rate is too fast, resulting in the crystal growth rate being too fast. A small particle size can exacerbate the occurrence of side reactions during the cycling process and easily adsorb moisture from the air, resulting in a large number of defects and crystalline water inside the grains, reducing the actual capacity and cycling life of the materials. However, oversized particles can hinder the transfer of Na^+^ within the material, affecting its rate performance. Continuing to extend the reaction time, the particle size begins to increase, indicating that the nucleation time slows down and the crystal can fully grow. When the reaction time is extended to 9 h, it is observed that many small cubes adhere to the surface of the large cube and fuse with it (Figure 14c). This is because the crystal is still in the growth stage and the growth process of the crystal overlaps with the formation process of new grains. However, when the reaction time is extended to 15 h, the surface of the sample becomes rougher and the cube corners collapse (Figure 14e). It may be that the reaction time is too long, and the already grown cube is damaged due to collision during high-speed stirring. In addition, prolonged exposure to high-temperature conditions after the full growth of crystals can lead to an increase in surface material dissolution. Therefore, the preferred reaction time is 12 h. The sample prepared under this condition has a more uniform distribution of grain size, a cleaner and smoother surface, and a reduced stacking situation.

**Figure 15:**
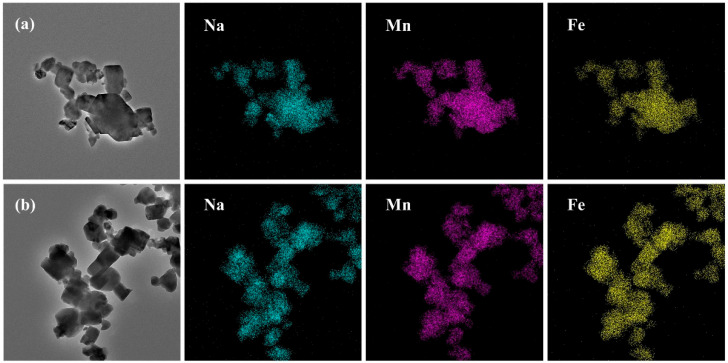
EDS-mapping diagrams of MnHCF prepared at different reaction times: (**a**) 3 h, (**b**) 12 h.

Figure 15 shows the EDS-mapping diagram of the sample at reaction times of 3 and 12 h. It can be seen from the graph that the 3 h sample is composed of particles of different sizes, and the particles have irregularity. The 12 h sample is mainly composed of cubic particles with a uniform particle size and certain dispersibility. Further investigation of the micromorphology and structure of the 3 h and 12 h samples using X-ray energy dispersive spectroscopy (EDS-mapping) shows that at a reaction time of 12 h, each element is uniformly distributed in the material cube particles, indicating that the prepared material has homogeneity. When the reaction time is 3 h, the aggregation phenomenon of various elements inside the larger sample particles is more obvious, and the distribution is not uniform enough.

**Figure 16:**
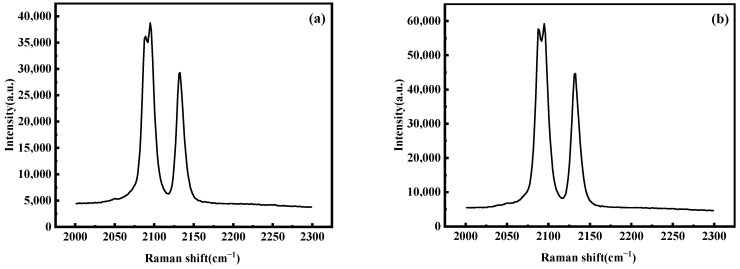
Raman spectra of MnHCF prepared at different reaction times: (**a**) 3 h, (**b**) 12 h.

Further testing was conducted of the Raman spectra of the material structure at reaction times of 3 h and 12 h, and the results are shown in Figure 16. From the graph, it can be seen that there are three distinct strong peaks: 2083 cm^−1^, 2089 cm^−1^, and 2126 cm^−1^, all of which are related to the (C≡N)^-^ carbon–nitrogen triple bond. The peaks at wave numbers 2089 cm^−1^ and 2126 cm^−1^ correspond to Fe^2+^-CN-Mn^2+^ and Fe^2+^-CN-Mn^3+^, respectively. However, no peaks related to Fe^3+^-CN-Mn^2+^ are observed in the Raman spectra at approximately 2180 cm^−1^, indicating that almost all Fe in the sample exists in the form of divalent Fe^2+^. Meanwhile, the peak strength of the sample with a reaction time of 12 h is higher, indicating a more complete material structure.

**Figure 17:**
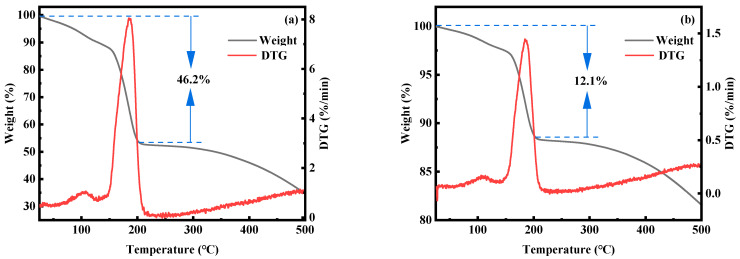
TGA spectra of MnHCF prepared at different reaction times: (**a**) 3 h, (**b**) 12 h.

To further analyze the water content in MnHCF samples, TGA thermogravimetric analysis was performed on materials with reaction times of 3 and 12 h. From Figure 17, it can be seen that the water content in the material with a reaction time of 3 h is about 49.5%, while the water content in the material with a reaction time of 12 h is about 12.7%. This indicates that extending the reaction time can effectively reduce the water content in the material during the preparation process.

The content of elements in materials with reaction times of 3 h and 12 h were detected and analyzed using inductively coupled plasma mass spectrometry (ICP-MS). The results are shown in Table 3. According to calculations, the molar ratios of Na, Mn, and Fe in materials with reaction times of 3 h and 12 h are 1.67/1/0.87 and 1.95/1/0.95, respectively. Based on the results of TGA analysis and calculation, the molecular formulas of the two materials can be obtained as follows: Na_1.67_Mn[Fe(CN)_6_]_0.87_·4.7H_2_O and Na_1.95_Mn[Fe(CN)_6_]_0.95_·1.3H_2_O. It can be concluded that a longer reaction time can effectively reduce the vacancy defects and crystal water content in MnHCF, while significantly increasing their sodium content.

**Figure 18:**
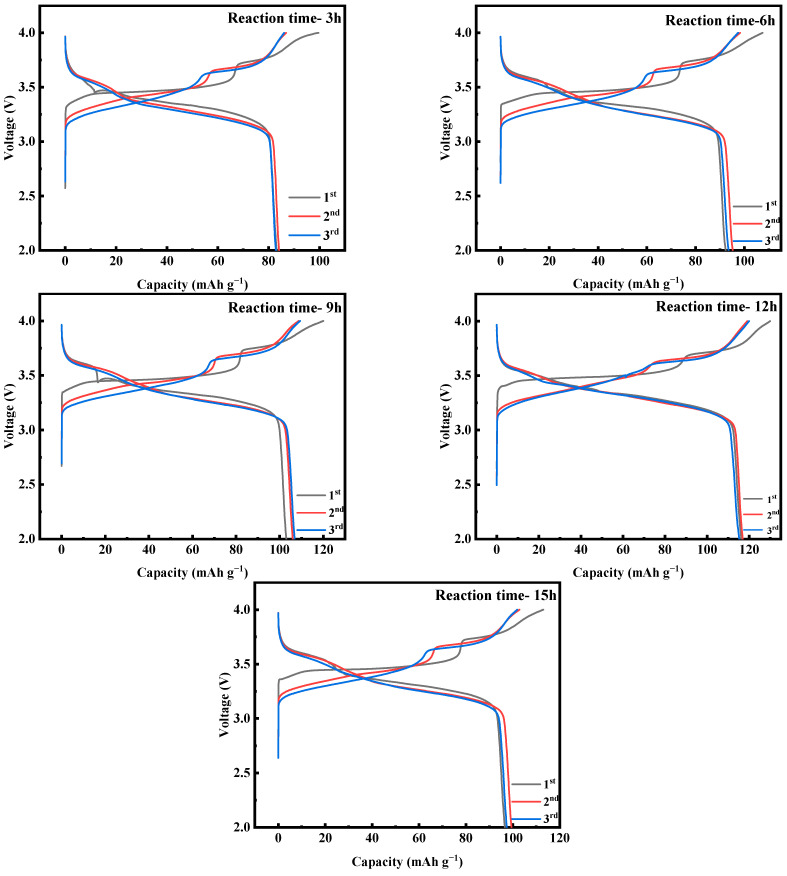
Charge–discharge curves of MnHCF prepared at different reaction times at 0.1 C for the first three cycles.

**Figure 19:**
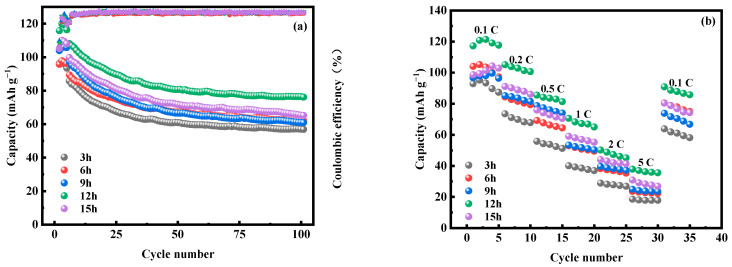
Cyclic and rate performances of MnHCF prepared at different reaction times: (**a**) cyclic performance and coulombic efficiency of MnHCF prepared at different reaction times at 0.2 C; (**b**) rate performance of MnHCF prepared at different reaction times.

Figure 18 shows the first three constant-current charging and discharging experiments of MnHCF electrode materials prepared at different reaction times under test conditions of 0.1 C and a voltage range of 2.0–4.0 V. From the graph, it can be seen that the initial discharge specific capacity of the electrode material prepared at a reaction time of 12 h is relatively large, reaching 116.3 mAh g^−1^. Figure 19a shows a comparison of the cyclic performance of MnHCF electrode materials prepared at different reaction times at 0.2 C. It can be seen that the sample exhibits a higher initial discharge specific capacity and a higher capacity retention rate when the reaction time is 12 h. The discharge capacity of the battery decreased to 76.2 mAh g^−1^ after 100 cycles, which is the highest among the five samples in terms of cycling performance. Figure 19b shows a comparison of the rate performance of samples prepared at different reaction times under different currents. It can be seen that the electrode discharge specific capacity prepared at 12 h of reaction time at different rates is higher than that of other samples, indicating a better rate performance. When the discharge capacity of the material is restored from 5 C to 0.1 C, it can recover to 75%, indicating good reversibility.

**Figure 20:**
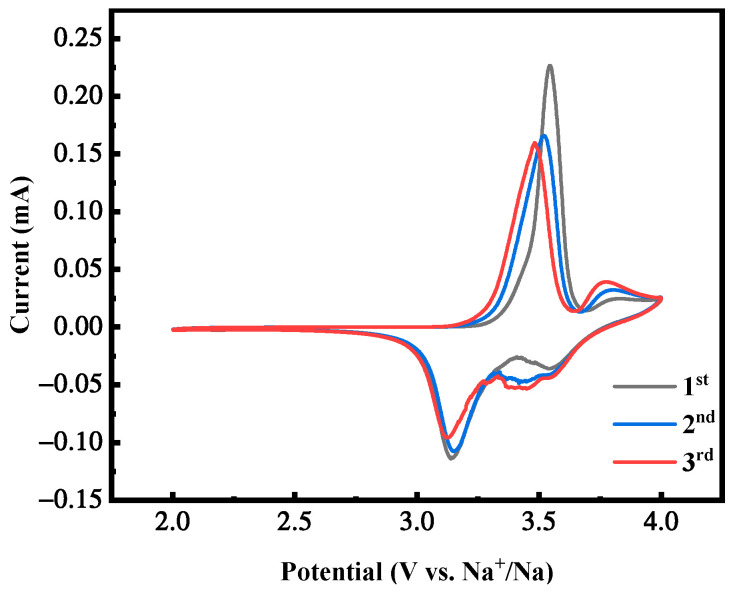
CV curves of MnHCF prepared at a reaction time of 12 h.

Figure 20 shows the cyclic voltammetry curve of the first three cycles of the MnHCF electrode with the best electrochemical performance under the reaction time of 12 h, with a voltage range of 2.0~4.0 V and a scanning rate of 0.1 mV/s. It can be seen that it has two pairs of redox peaks. The area of this redox peak is much higher than the sample tested earlier. Meanwhile, as the cycle progresses, there is a good overlap in the curves, indicating that the electrochemical reactions of the two cathodes have high reversibility and maintain relatively stable electrochemical activity, corresponding to and consistent with the plateau of their constant-current charge–discharge curves.

**Figure 21:**
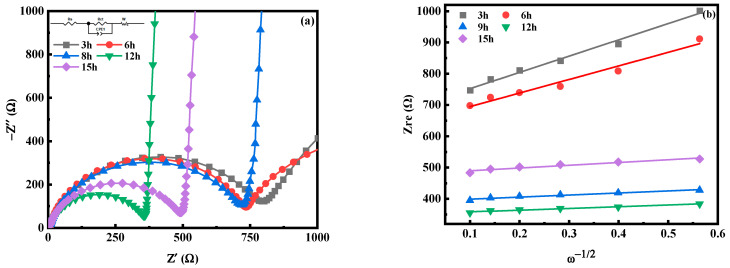
EIS spectra of MnHCF prepared with different reaction times: (**a**) EIS spectra; (**b**) linear relationship of Z_re_ and ω^−1/2^ of MnHCF electrodes prepared with different reaction times.

Figure 21 is the electrochemical impedance spectroscopy (EIS) test diagram of MnHCF electrodes prepared with different reaction times, and the illustration in Figure 21a is the fitted equivalent circuit diagram. The electrochemical impedance spectra of electrodes prepared from different samples are composed of oblique lines in the low-frequency region and semicircles in the high-frequency region, belonging to typical Nyquist and Warburg impedances. The semicircle region corresponds to the electron transfer impedance R_ct_ at the interface between the electrode material and the electrolyte. The R_ct_(297.5Ω) of the electrode prepared at a reaction time of 12 h is smaller than that of other materials, indicating that Na^+^ and e^−^ transport faster at the interface between the electrode material and electrolyte and have a better rate performance. The diffusion coefficient of Na^+^ in electrodes prepared with different materials can also be calculated, as shown in Table 4. The D_Na_ of the electrode material prepared at a reaction time of 12 h is significantly higher than that of other materials, indicating the desorption kinetics and diffusion rate of Na^+^ have been improved.

Through the above analysis, it can be concluded that, by controlling various reaction conditions during the coprecipitation process, MnHCF materials with a higher specific capacity can be prepared. While effectively improving the material’s specific capacity, it can also exhibit a relatively good capacity retention under high currents. This contributes to the growth law of crystals: under suitable raw material ratios, low concentrations, and longer reaction environments, the reaction rate can be effectively reduced, making the crystal growth rate less than the nucleation rate. Continuous and recrystallization can be carried out, thereby reducing the lattice defects and lattice water formed by the crystal, demonstrating a relatively better electrochemical performance.

## 3. Experimental Details

### 3.1. Materials

Sodium sulfate (Na_2_SO_4_, AR), manganese sulfate monohydrate (MnSO_4_∙H_2_O, AR), sodium ferrocyanide (Na_4_Fe(CN)_6_, ≥98%), and anhydrous alcohol (C_2_H_5_OH, AR) were purchased from Sinopharm Chemical Reagent Co. or Aladdin Industrial Inc, Ltd., Shanghai, China. All the chemicals were used without purification.

### 3.2. Material Synthesis

**Figure 22:**
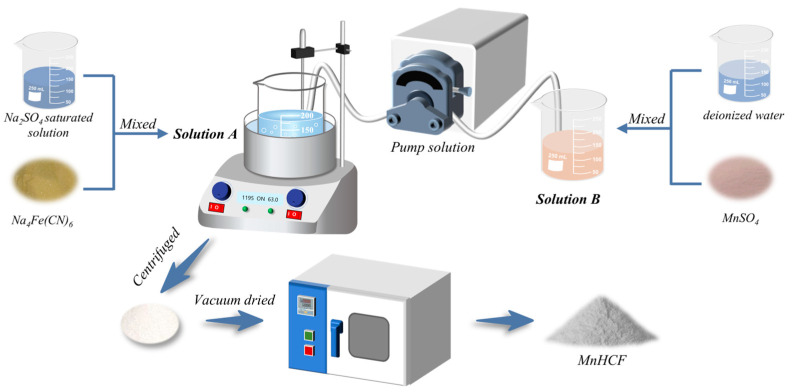
Schematic diagram of the preparation process of MnHCF.

The MnHCF material was prepared using the coprecipitation method, and the preparation process is shown in Figure 22. Firstly, 3 mmol of sodium ferrocyanide (Na_4_Fe(CN)_6_) was dissolved in 100 mL of saturated Na_2_SO_4_ solution (solution A). Secondly, a certain amount of manganese sulfate monohydrate (MnSO_4_∙H_2_O) was dissolved in 100 mL of deionized water in Fe:Mn molar ratios of 1:0.5, 1:1, 1:1.5, 1:2, and 1:2.5 (Solution B). After determining the optimal Fe-Mn molar ratio, 0.01 mol/L, 0.02 mol/L, 0.03 mol/L, and 0.04 mol/L MnSO_4_ solutions were prepared separately for experiments. Thirdly, the reaction temperature was set to 85 °C. Solution B was controlled to be added to solution A at different rates, resulting in reaction times of 4 h, 6 h, 8 h, 10 h, and 12 h, respectively. After the reaction was completed, it was aged for 2 h. The obtained samples were centrifuged, washed with deionized water and ethanol several times, and then vacuum-dried at 110 °C for 18 h. The relevant reaction formula is as follows:

Na_4_Fe(CN)_6_ + Mn^2+^ + H_2_O → Na_x_Mn[Fe(CN)_6_]_1−y_□y nH_2_O + e^−^ (0 < x < 2, 0 < y < 1)

### 3.3. Material Characterization

The structure and phase were analyzed using an X-ray diffractometer (D/Max2500) produced by Rigaku (Tokyo, Japan). A field emission scanning electron microscope (SEM) produced by Hitachi SU8020 (Tokyo, Japan) was used to analyze the structure, morphology, and elements of manganese oxides. Raman spectra were conducted on a LabRAM HR800 (Horiba JobinYvon, Longjumeau, France) using an Ar+ laser with a wavelength of 532 nm. Elemental compositions of Na, Fe, Mn, C, N, and H were determined using a combination of the ICP-OES SPECTRO ARCOS MV (SPECTRO, Kleve, Germany) and an elemental analyzer (vario EL III, Langenselbold, Germany).

### 3.4. Electrochemical Measurements

The electrochemical performance of the prepared MnHCF products was studied with button cells. The active substances, acetylene black and polyvinylidene fluoride (PVDF), were weighed at a mass ratio of 7:2:1 and mixed and grounded to produce a black paste slurry. Then, the black paste slurry was uniformly coated on aluminum foil and dried in a vacuum-drying oven (110 °C) for 12 h. Next, the aluminum foil was taken out and cut into a positive membrane with a diameter of 12 mm. Finally, the CR2032 button battery was assembled in a dry glove box filled with high-purity argon (99.999%) (H_2_O ≤ 0.5 ppm), MnHCF was the positive electrode, the sodium metal sheet was the negative electrode, the glass fiber (GF/D) was the diaphragm, and 1 M NaPF_6_ in ethylene carbonate (EC)/diethyl carbonate (DEC) (1:1 in volume) was the electrolyte. The constant-current charge–discharge performance, magnification performance, and cycle performance of the battery were tested using the CT-4008T-5V battery test system, and the voltage range was 2–4 V. The charge–discharge experiment was carried out at a constant temperature of 25 °C. The cyclic voltammetry of the battery was conducted using a CH1660D electrochemical workstation. The test voltage range was 2~4 V, and the scanning speed was 0.1 mV/s. The AC impedance spectrum, fitting circuit, AC impedance, and other parameters of the battery were tested. The test voltage range was 2~4 V, and the frequency range was 10^−2^~10^5^ Hz.

## 4. Conclusions

The coprecipitation method is a highly feasible method for achieving the industrial production of Prussian blue analogs. Among them, regulating various synthesis conditions plays an important role in obtaining materials with excellent properties. In this study, the influence of different reaction conditions on the electrochemical performance of cathode materials for sodium-ion batteries has been systematically studied. The results showed that different reaction conditions led to different structures and electrochemical properties. When Fe:Mn = 1:2, c(Mn^2+^) = 0.02 mol/L, and the reaction time was 12 h, the resulting material exhibited a high sodium content, low interstitial water molecular content, and Fe(CN)_6_ defects. In addition, the electrode prepared by this sample achieved a high discharge specific capacity of 121.9 mAh g^−1^, with a capacity retention rate of 65% after 100 cycles at 0.2 C. It has the best cycling stability, rate performance, and reversible performance among all the prepared samples.

In summary, this work has prepared MnHCF materials with a high charge–discharge capacity and good low current cycling performance by regulating a series of reaction conditions. Compared with previous work, this work obtained MnHCF materials with excellent electrochemical performance by simply controlling the crystallization process and systematically studied the reasons for the influence of different reaction conditions on the electrochemical performance of the materials. This experimental process is environmentally friendly, cost-effective, and has a high yield, making it suitable for large-scale production. It effectively promotes the commercial application of this material in the positive electrode material of sodium-ion batteries.

## Figures and Tables

**Table 1:** The fitting results of impedance spectra and the calculated D_Na_ values of different MnHCF prepared with different Fe-Mn molar ratios.

Samples	R_s_ (Ω)	R_ct_ (Ω)	D_Na_ (cm^2^s^−1^)
Fe:Mn = 1:0.5	2.77	514	1.55 × 10^−14^
Fe:Mn = 1:1	7.73	1530	4.20 × 10^−14^
Fe:Mn = 1:1.5	3.53	1028	1.32 × 10^−14^
Fe:Mn = 1:2	4.18	829	7.02 × 10^−14^
Fe:Mn = 1:2.5	8.34	346	8.56 × 10^−13^

**Table 2:** The fitting results of impedance spectra and the calculated D_Na_ values of different MnHCF materials.

Samples	R_s_ (Ω)	R_ct_ (Ω)	D_Na_ (cm^2^s^−1^)
c(Mn^2+^) = 0.01 mol/L	4.20	654.6	3.81 × 10^−13^
c(Mn^2+^) = 0.02 mol/L	4.60	480.1	3.97 × 10^−13^
c(Mn^2+^) = 0.03 mol/L	4.25	483.3	3.01 × 10^−13^
c(Mn^2+^) = 0.04 mol/L	6.13	583.8	2.02 × 10^−13^

**Table 3:** Element analysis of MnHCF materials prepared with reaction times of 3 h and 12 h.

Samples	Element Ratio		
	Na	Mn	Fe
3 h	1.67	1	0.87
12 h	1.95	1	0.95

**Table 4:** The fitting results of impedance spectra and the calculated D_Na_ values of different MnHCF materials.

Samples	R_s_ (Ω)	R_ct_ (Ω)	D_Na_ (cm^2^s^−1^)
3 h	2.17	639.1	2.52 × 10^−13^
6 h	2.71	606.9	3.63 × 10^−13^
9 h	8.26	351.3	1.61 × 10^−11^
12 h	10.65	297.5	2.25 × 10^−11^
15 h	8.27	435.8	8.80 × 10^−12^

## Data Availability

The data presented in this study are available on request from the corresponding author. The data are not publicly available due to [our laboratory’s policies or confidentiality agreements].
